# Biofunctionalization of ADA‐GEL Hydrogels Based on the Degree of Cross‐Linking and Polymer Concentration Improves Angiogenesis

**DOI:** 10.1002/adhm.202500730

**Published:** 2025-03-17

**Authors:** Stefanie Heltmann‐Meyer, Rainer Detsch, Jonas Hazur, Lasse Kling, Sabrina Pechmann, Rajkumar Reddy Kolan, Justus Osterloh, Aldo R. Boccaccini, Silke Christiansen, Carol I. Geppert, Andreas Arkudas, Raymund E. Horch, Dominik Steiner

**Affiliations:** ^1^ Department of Plastic and Hand Surgery University Hospital of Erlangen Friedrich‐Alexander‐Universität Erlangen‐Nürnberg (FAU) 91054 Erlangen Germany; ^2^ Institute of Biomaterials University of Erlangen‐Nürnberg 91058 Erlangen Germany; ^3^ Institute for Nanotechnology and Correlative Microscopy gGmbH (INAM gGmbH) 91301 Forchheim Germany; ^4^ Department for Correlative Microscopy and Materials Data Fraunhofer Institute for Ceramic Technologies and Systems (IKTS) 91301 Forchheim Germany; ^5^ Department of Plastic and Hand Surgery University of Freiburg Medical Center 79106 Freiburg Germany; ^6^ Fachbereich Physik Freie Universität Berlin (FU Berlin) 14195 Berlin Germany; ^7^ Institute of Pathology University Hospital of Erlangen Friedrich‐Alexander‐Universität Erlangen‐Nürnberg (FAU) 91054 Erlangen Germany; ^8^ Comprehensive Cancer Center Erlangen‐EMN (CCC ER‐EMN) University Hospital Erlangen FAU Erlangen‐Nuremberg 91054 Erlangen Germany; ^9^ Department of Hand Plastic Reconstructive and Burn Surgery BG Trauma Clinic University of Tübingen 72076 Tübingen Germany

**Keywords:** ADA‐GEL, angiogenesis, biofunctionalization, hydrogel, tissue engineering

## Abstract

The creation of bioartificial tissues is a promising option for the reconstruction of large‐volume defects. The vascularization of tissue engineering constructs, as well as the material properties of the carrier matrix, are important factors for successful clinical application. In this regard, hydrogels are promising biomaterials, providing an extracellular matrix‐like milieu that enables the possibility of cell transplantation and de novo tissue formation. Furthermore, biofunctionalization allows for a certain fine‐tuning of angiogenic properties. This study aims to investigate vascularization and tissue formation of highly cross‐linked alginate dialdehyde (ADA) and gelatin (GEL). This highly cross‐linked network is created using a dural cross‐linking mechanism combining ionic (Ca^2+^ ions) and enzymatic (human transglutaminase (hTG)) cross‐linking, resulting in reduced swelling and moderate degradation rates. Vascularization of the ADA‐GEL‐hTG constructs is induced surgically using arteriovenous (AV) loops. Biocompatibility, tissue formation, and vascularization are analyzed by histology and X‐ray microscopy. After only 2 weeks, vascularization of the ADA‐GEL‐hTG constructs is already present. After 4 weeks, both de novo tissue formation and vascularization of the ADA‐GEL‐hTG matrix increase. In conclusion, ADA‐GEL‐hTG‐based hydrogels are shown to be promising scaffold materials for tissue engineering applications.

## Graphical abstract

The biocompatibility of alginate dialdehyde (ADA) and gelatin (GEL) hydrogels cross‐linked with human transglutaminase (hTG) is demonstrated in vivo with a high degree of stability and angiogenic potential. The additional enzymatic cross‐linker hTG and the increased concentration improve stability and angiogenic potential. The X‐ray microscopy (XRM) is employed for the first time. It allows visualization and precise analysis of the vascular network, provided that effective perfusion of the vessels is achieved by the contrast agent.

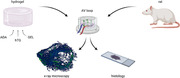

## 1 Introduction

To identify suitable biomaterials for tissue regeneration applications, it is necessary to ascertain that they fulfill a number of specific requirements. Besides enabling an extracellular matrix‐like milieu, good biocompatibility, and the possibility of biodegradation, the biomaterials should support vascularization. Regarding clinical application, vascularization of tissue engineering constructs is the bottleneck. Only a sufficient microvascular endothelial network connected to the host organism allows for cell survival and thereby de novo tissue formation. Polysaccharides such as alginate polymers, which are obtained from algae, are common biomaterials.^[^ ^1^ ^]^ They are already widely used in the food industry and in medicine.^[^ ^2^ ^]^ For medical applications, alginate is used primarily in wound care and, due to its ability to form hydrogels, achieves good results in wound healing.^[^ ^3^ ^]^ Alginate is often used as a soft hydrogel, but can also be transformed into a very firm matrix that is able to withstand greater loads.^[^ ^4^ ^]^ It is also characterized by its good biocompatibility.^[^ ^5^ ^]^ Moreover, alginate affords the possibility of forming it into a desired 3D shape, that it can retain by cross‐linking it with calcium chloride (CaCl_2_) and in addition also offers a rigid structure independent of temperature. This provides a wide range of applications. However, low biodegradability and the absence of cell adhesion motifs are major disadvantages of pure alginate.^[^ ^6^ ^]^ Through modification by means of partial oxidation, alginate can be covalently bonded to gelatin (GEL), resulting in the so‐called alginate dialdehyde–gelatin (ADA‐GEL).^[^ ^7^ ^]^ The cross‐linking of alginate and gelatin has been optimized, resulting in a complete conversion from sol to gel.^[^ ^8^ ^]^ The inner pore structure of ADA‐GEL was revealed by cryo‐SEM images. Depending on the strength of the ionic Ca^2+^ cross‐linking, a pore area between 1.5 ± 0.9 and 3.4 ± 0.2 µm^2^ was found.^[^ ^9^ ^]^

The coupled gelatin offers integrin‐binding sequences (RGD) to which cells can better adhere, and thus promotes biocompatibility as well as angiogenesis.^[^ ^10^ ^]^ Gelatin is a well‐known biomaterial for tissue engineering applications, supporting de novo tissue formation and angiogenesis.^[^ ^11^ ^]^ However, gelatin itself degrades quite rapidly, which can be detrimental during prolonged periods of application.^[^ ^12^ ^]^ ADA‐GEL is a hydrogel that has already been successfully tested in research.^[^ ^13^ ^]^ ADA‐GEL with a lower concentration (2.5/2.5% wv) has also been investigated in vivo in AV loops, in the form of microcapsules demonstrating good vascularization and biocompatibility properties.^[^ ^13a^ ^]^ In our study, a higher concentration and different composition of ADA‐GEL (3.75/7.5% wv) was used, which was evaluated in a previous study.^[^ ^14^ ^]^ In this work, it was shown that CaCl_2_ and additionally microbial transglutaminase (mTG) increase stiffness, swelling behavior, and degradation rates. Besides that, cross‐linking with mTG leads to a further transformation and the conversion of the gelatin‐containing components into a solid state.^[^ ^14^ ^]^ Transglutaminases occur naturally, for example in the blood, where they act as factor XIII in clotting by means of fibrin.^[^ ^15^ ^]^ The reason for the application of this double cross‐linking was to increase the stability of the ADA‐GEL hydrogels without affecting their biocompatibility, for example by using additional chemical cross‐linkers such as genipin.^[^ ^12^ ^]^

For the adequate vascularization of the ADA‐GEL‐hTG constructs, we used the well‐established rat arteriovenous loop model (AV loop).^[^ ^11^, ^16^ ^]^ Forming an arteriovenous fistula between the saphenous artery and vein allows for a reliable vascularization of biomaterials upon 2 to 4 weeks after implantation. In addition to that, the AV loop model is a highly standardized in vivo model for investigating biocompatibility, biodegradation, de novo tissue formation, and vascularization of innovative biomaterials. For our study, we have chosen a short (2 weeks) and a longer (4 weeks) implantation period in order to discriminate between early and late biomaterial–host interactions. Besides conventional histology, we established X‐ray Microscopy (XRM) as a new method in order to describe vascularization originating from the AV loop.

## 2 Results

### 2.1 Postoperative Follow‐Up, Construct Stability and Macroscopic Appearance

All rats tolerated the surgery and implantation period well without wound healing disturbances or rejection reactions. After explantation, five of six constructs (83.3%) in the 2‐week group and four of six (66.7%) in the 4‐week group were identified as patent and were included in the study.

**Figure 1:**
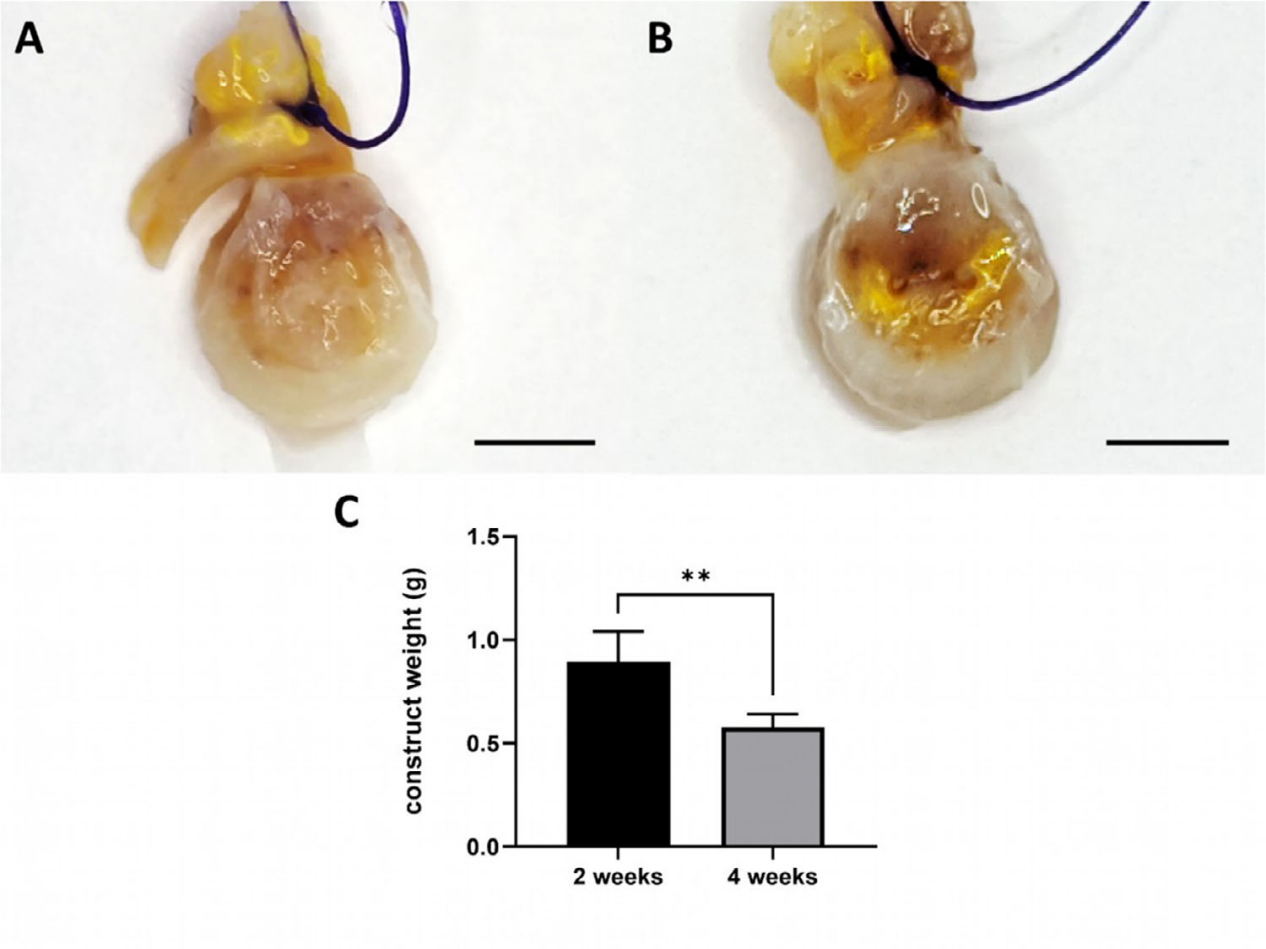
Macroscopic appearance and construct weight. The explants were opaque and the vessels appeared yellow‐colored upon the perfusion with Microfil after 2 weeks A) and after 4 weeks B). The hydrogel construct weight was significantly lower after 4 weeks C). Scale bar = 5 mm (A, B). Quantification of the construct weight is shown as mean ± SD. 2‐week group *n* = 5 specimen and 4‐week group *n* = 4 specimen. Shapiro–Wilk normality test followed by *t*‐test was used for statistical analysis. Statistically significant differences are indicated for ^**^ *p* ≤ 0.01.

The explants were soft, and deformable but stable, with yellow‐colored vessels due to the contrast agent (**Figure** **1**). The weight of the constructs decreased significantly from the 2 ‐ to the 4‐week group (1.62 ± 0.13 g vs 1.36 ± 0.12 g; *p* = 0.004) (Figure 1).

### 2.2 Tissue Formation and Immune Response

**Figure 2:**
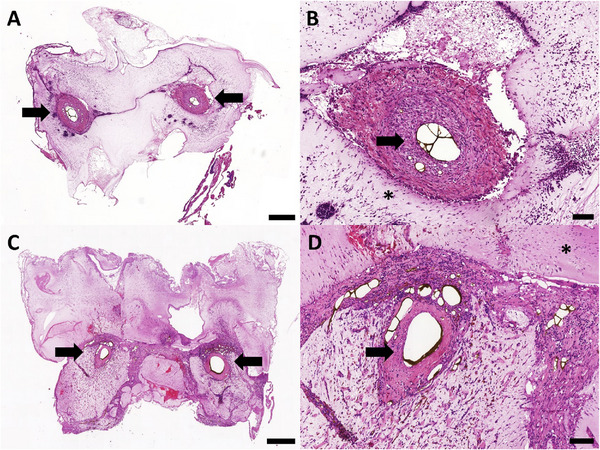
Histological overview with detailed images A–D). H&E staining was performed and microphotographs were taken of the 2‐ (A, B) and 4‐week (C, D) groups. Newly formed vascularized tissue originating from the AV loop was found in all groups. The ADA‐GEL‐hTG matrix is indicated by the asterisks (^*^) and the AV loop by the bold arrows. Scale bar = 500 µm (A, C) and 100 µm (B, D). Images based on scans with PANNORAMIC 250 Flash scanner and prepared using CaseViewer 2.4, 0.23 µm pixel^−1^ (3DHISTECH, Budapest, Hungary).

In all constructs, newly formed and highly vascularized tissue was found (**Figure** **2**). Newly formed connective tissue was found after 2 weeks and 4 weeks. This was mainly located directly around the main loop vessels. After 4 weeks, the connective tissue also began to extend further into the periphery of the construct (Figure 2D).

**Figure 3:**
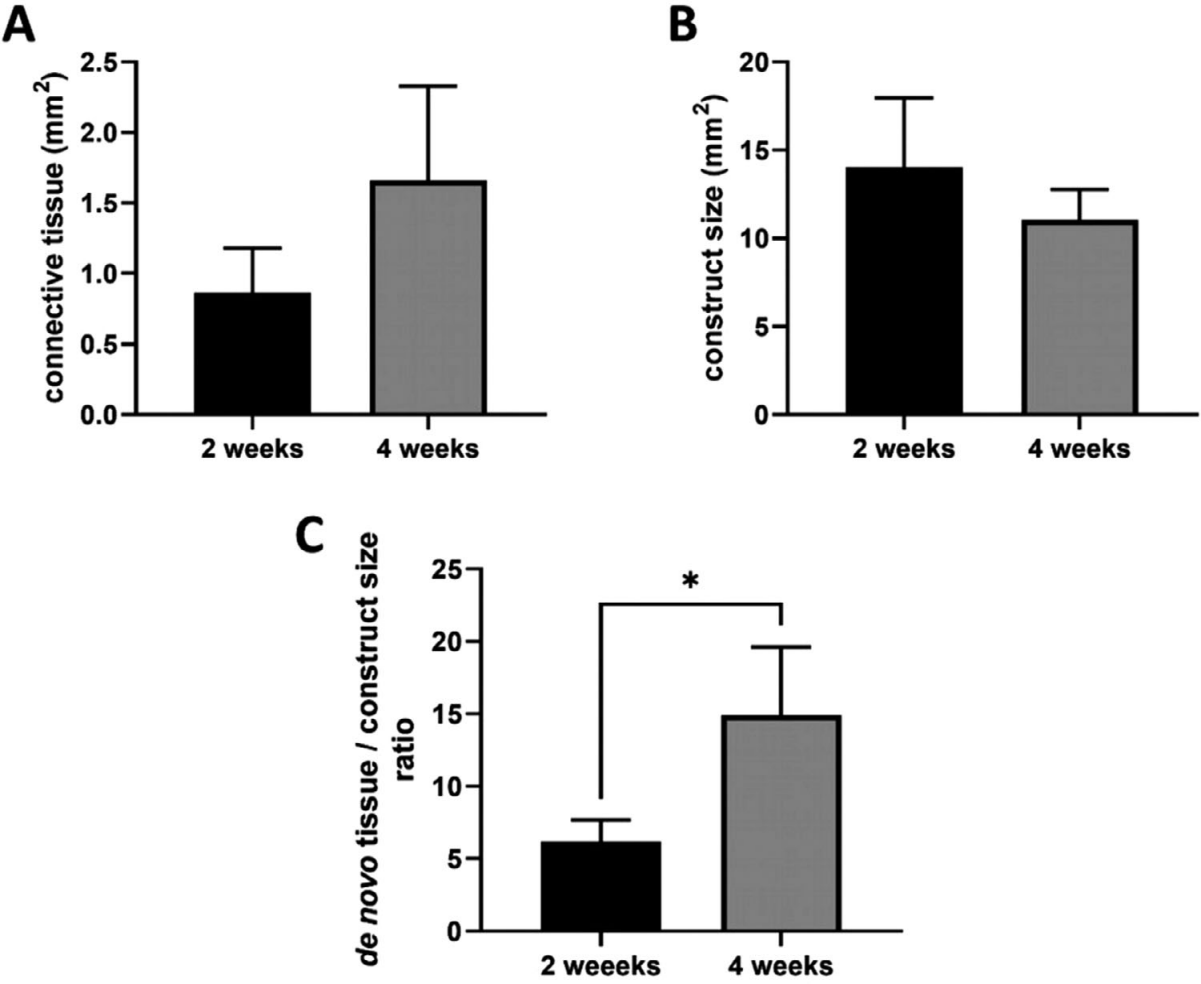
Quantification of tissue formation A), construct size B), and de novo tissue/construct size ratio C). Although the connective tissue area and construct size did not change in a statistically significant way (A, B), there was clear remodeling in the 4‐week constructs. The de novo tissue/construct size ratio increased significantly after 4 weeks (C). Quantification data of n = 5 rats (2‐week group) and n = 4 rats (4‐week group) is shown as mean ± SD from 1 representative histological cross‐section. Shapiro–Wilk normality test followed by *t*‐test was used for statistical analysis. Statistically significant differences are indicated for ^*^ *p* ≤ 0.05.

The newly formed tissue was predominantly located around the AV loop with a trend of more connective tissue after 4 weeks compared to the 2‐week group (1.66 ± 0.67 mm^2^ vs 0.86 ± 0.32 mm^2^; n.s.) (**Figure** **3**A). Comparing the construct size between the 2‐ and the 4‐week group, no statistically significant differences were detected (14.03 ± 3.93 mm^2^ vs 11.02 ± 1.76 mm^2^ n.s.) (Figure 3B). The ratio between the connective tissue area and the construct size, as a surrogate parameter for remodeling, increased significantly from 2 to 4 weeks (6.18 ± 1.50 vs 14.93 ± 4.68; *p*  =  0.01) (Figure 3C).

**Figure 4:**
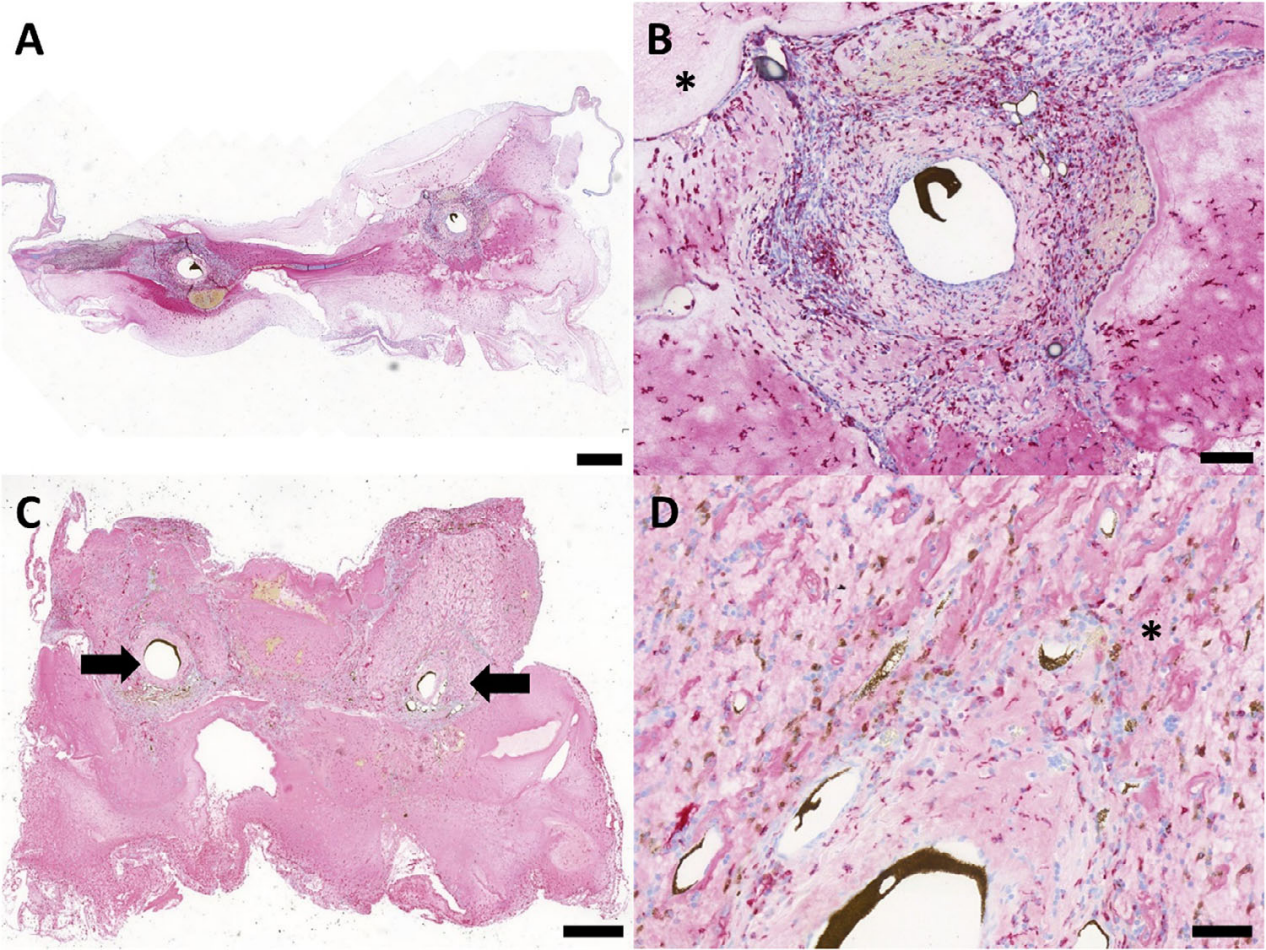
CD68 immunostaining. In order to visualize CD68‐positive cells, an anti‐CD68 antibody was used after 2 A,B) and 4 weeks C,D) implantation time. The bold arrows indicate the AV loop, and the asterisks (^*^) are the ADA‐GEL‐hTG matrix. Macrophages are colored in red. Scale bar = 500 µm (A, C) and 100 µm (B, D). Images based on scans with PANNORAMIC 250 Flash scanner and prepared using CaseViewer 2.4, 0.23 µm pixel^−1^ (3DHISTECH, Budapest, Hungary).

To determine the immune response toward the matrix, macrophages were highlighted using CD68 staining (**Figure** **4**). No multinuclear giant cells were found. CD68‐positive cells were predominantly located in the highly vascularized construct parts as well as in the interface between the connective tissue and the ADA‐GEL‐hTG matrix. Moreover, macrophages can be observed in non‐vascularized parts of the ADA‐GEL‐hTG matrix.

**Figure 5:**
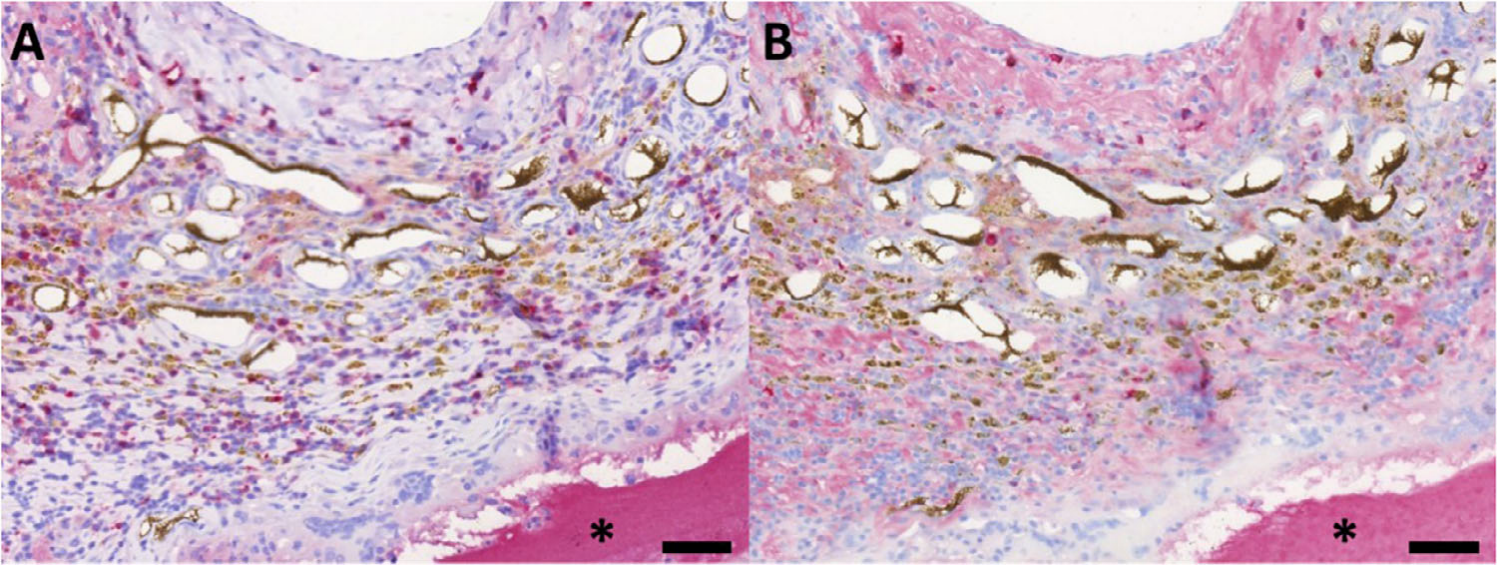
CD86 and CD163 immunostaining. To visualize proinflammatory (M1) macrophages an anti‐CD86 antibody was used A). Conversely, anti‐inflammatory M2 macrophages were detected with an anti‐CD163 antibody B). The asterisks (^*^) indicate the ADA‐GEL‐hTG matrix. M1 or M2 Macrophages are colored in red. Scale bar = 500 µm]. Images based on scans with PANNORAMIC 250 Flash scanner and prepared using CaseViewer 2.4, 0.23 µm pixel^−1^ (3DHISTECH, Budapest, Hungary).

Additionally, CD86 and CD163 immunostainings were carried out in the 4‐week group to differentiate between the pro – (M1) and anti‐inflammatory (M2) subtypes. We were able to detect M1 and M2 macrophage subtypes in the specimen without a tendency toward the pro‐ or anti‐inflammatory subtype (**Figure** **5**).

### 2.3 Surgically Induced Angiogenesis and Construct Vascularization

**Figure 6:**
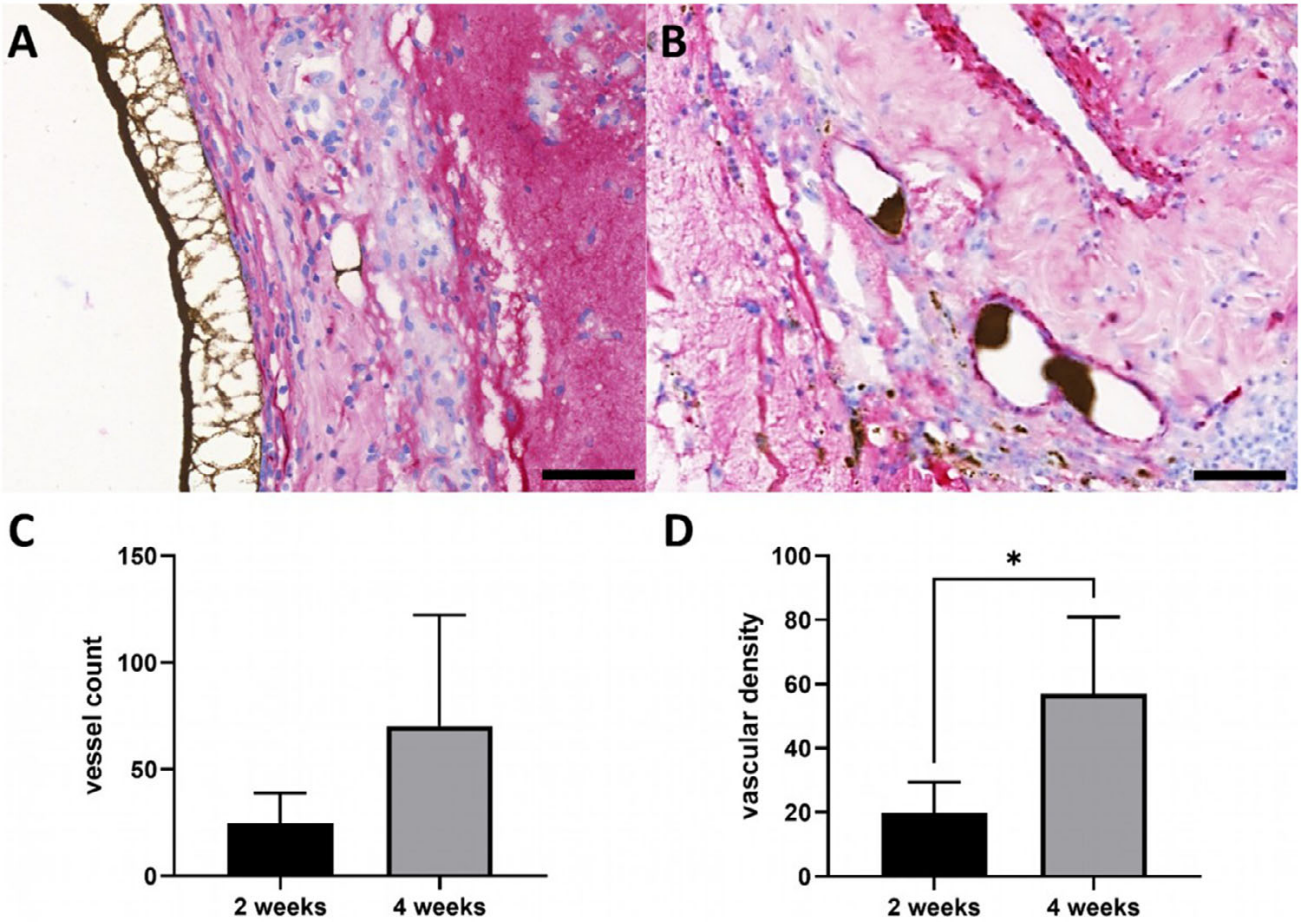
α‐SMA staining depicting smooth muscle cells in the media layer and quantification of vascularization. Smooth muscle cells were red‐stained using an anti‐α‐SMA antibody, with A) being the 2‐week group, and B) being the 4‐week group. The vessel count revealed no significant changes from 2 to 4 weeks implantation time C), but in the number of vessels per connective tissue area D). Scale bar = 50 µm. Images based on scans with PANNORAMIC 250 Flash scanner and prepared using CaseViewer 2.4, 0.23 µm pixel^−1^ (3DHISTECH, Budapest, Hungary). Quantification data of n = 5 rats (2‐week group) and n = 4 rats (4‐week group) is shown as mean ± SD from 1 representative histological cross‐section. Shapiro–Wilk normality test followed by *t*‐test was used for statistical analysis. Statistically significant differences are indicated for ^*^ *p* ≤ 0.05.

As previously described, we were able to prove that newly formed vessels originated from the AV loop in all constructs (Figure 2A–D). The Microfil was located inside the vessels and no Microfil was found in the newly formed tissue or ADA‐GEL‐hTG matrix as a surrogate parameter for the vessel integrity. Using α‐SMA staining, we were able to demonstrate that most of the newly formed vessels contained smooth muscle cells in their media layer (**Figure** **6**). Calculating the vessel number per histological cross‐section did not reveal a statistically significant difference between the 2‐week and 4‐week groups (24.5 ± 14.48 vs 70.25 ± 52.11; n.s.). In addition to this, the vessel density, calculated as vessel number per connective tissue area (in µm2), indicated statistically significant differences (19.81 ± 9.65 µm^−2^ vs 56.95 ± 23.96 µm^−2^; *p* = 0.03) (Figure 6C,D).

**Figure 7:**
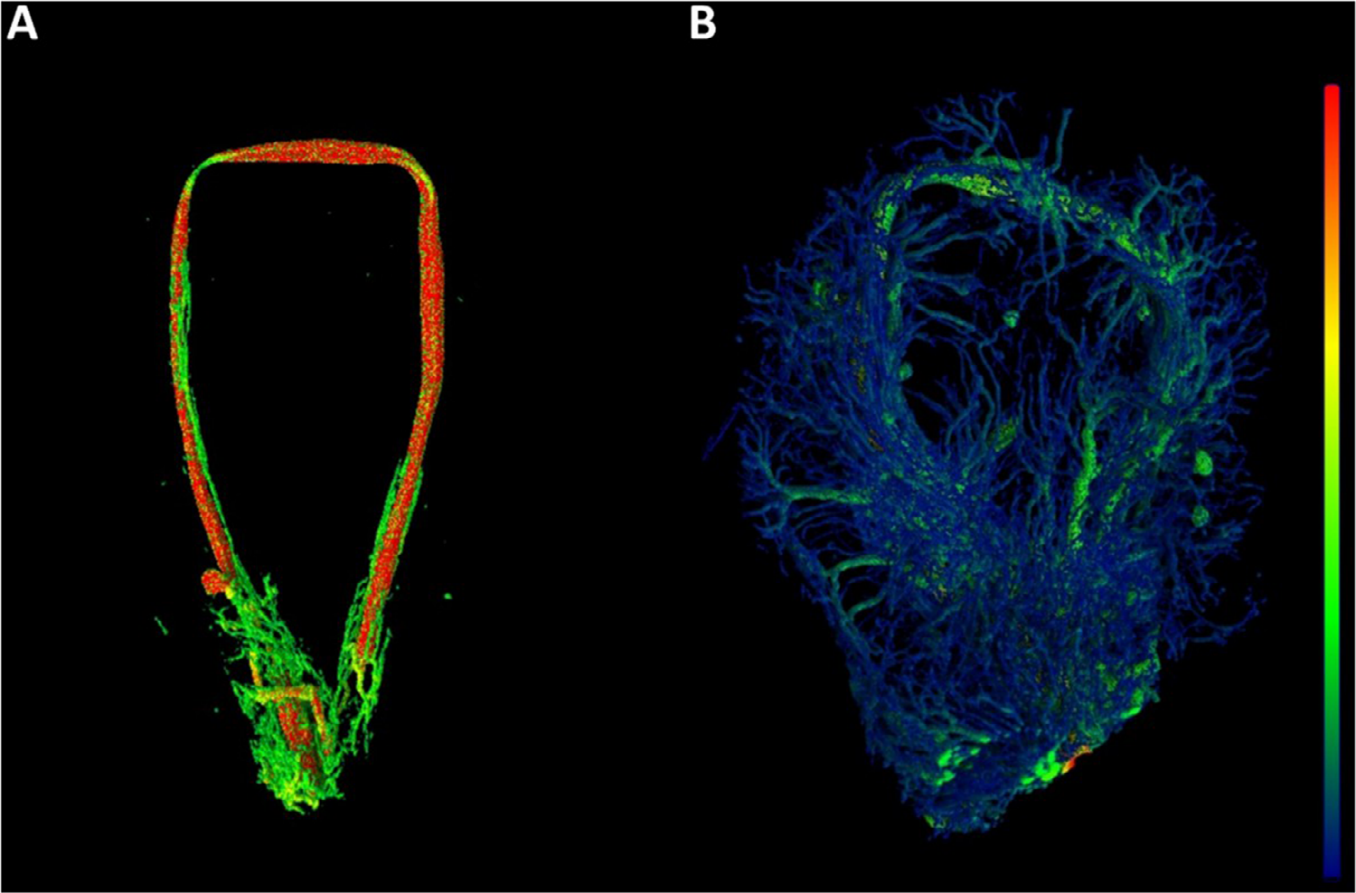
X‐ray microscopy (XRM). After 2 A) and 4 weeks B), the constructs were perfused with Microfil and scanned by XRM. The 3D reconstructed images are color‐coded according to vessel thickness from thin (blue) to thick (red). The resulting 3D image data was analyzed with respect to the thickness and length of the vessels. After 4 weeks, an increase in vessel formation was detected when compared with the 2‐week group.

For a more detailed analysis of new vessel formation, the explants were examined by X‐ray microscopy (XRM). Since histology only allows a limited spatial view of vascularization, XRM allows for a more holistic approach. Corresponding to the macroscopic and histological findings, the XRM revealed 5 out of 6 patent AV loops after 2 weeks and 4 out of 6 after 4 weeks. Of those, one construct had to be excluded due to incomplete perfusion. After 2 weeks, newly formed vessels originating from the AV loop were found in the XRM reconstructions (**Figure** **7**A). After 4 weeks, a larger and denser vessel network was found (Figure 7B).

**Figure 8:**
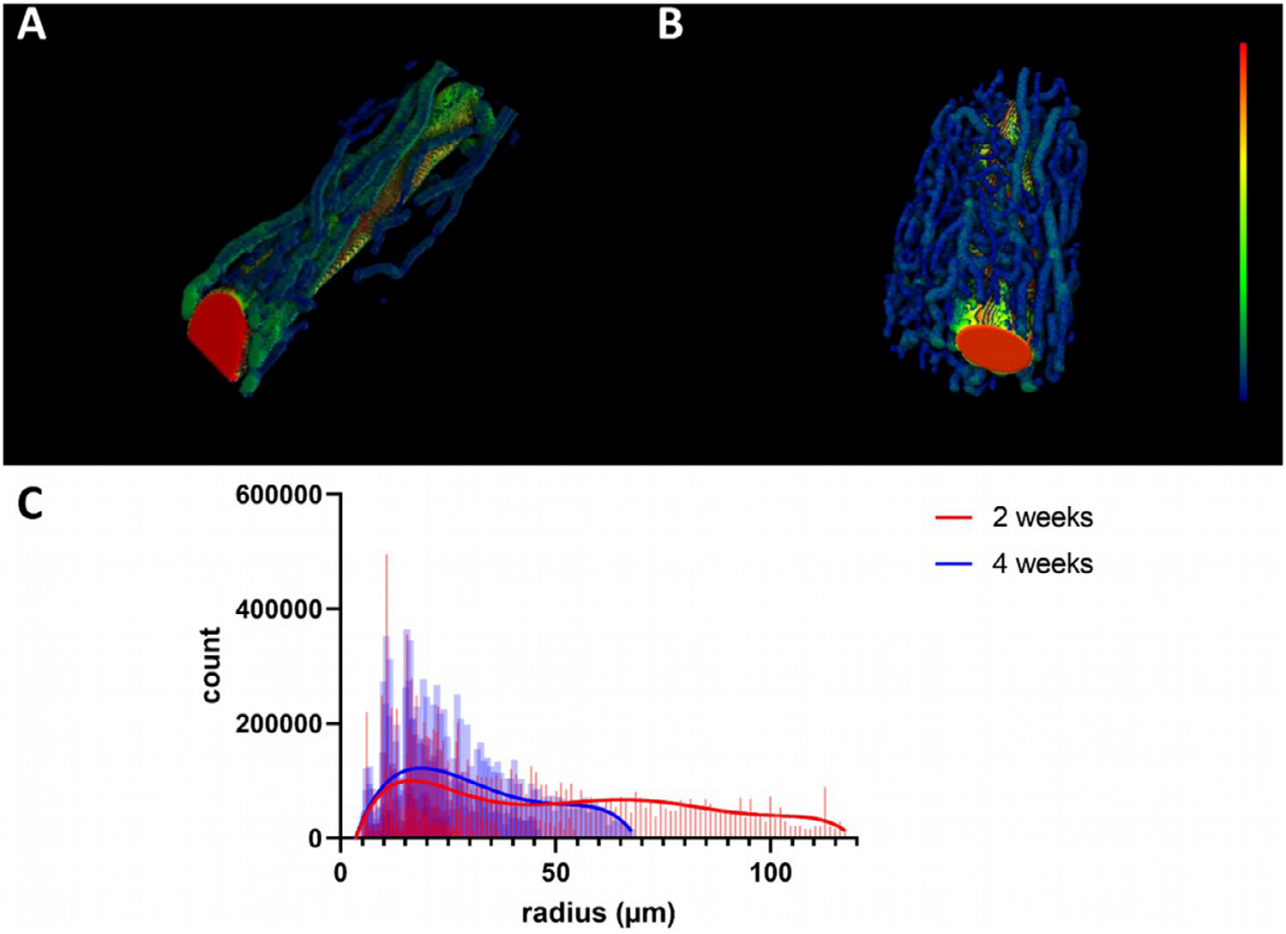
Analysis of vessel thickness and their distribution in the XRM. After 4 weeks, XRM revealed a denser vessel network B) compared to the 2‐week group A) (color‐coded according to vessel thickness from thin (blue) to thick (red)). After 4 weeks, the newly formed vessels displayed a larger diameter C). Data plotted as non‐linear regression; sixth order polynomial. The 2‐week group is shown in red, and the 4‐week group in blue.

A more detailed look at the newly formed vessels originating from the AV loop revealed a denser vessel network after 4 weeks (**Figure** **8**A,B). Analyzing the radius of the newly formed vessels, we were able to demonstrate statistically significant larger vessels in the 4‐week group compared to the 2‐week group (77.06 ± 61.0 vs 53.31 ± 33.60 µm; *p* ≤ 0.01) (Figure 8C).

**Figure 9:**
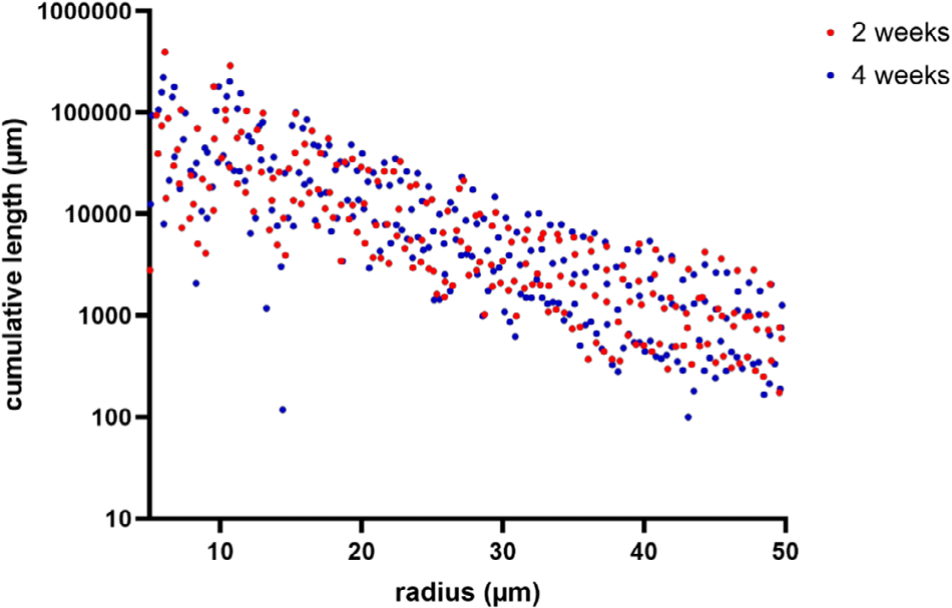
Cumulative vessel length in the XRM. Vessel length is calculated as a dependency of the radius from 5 up to 50 µm. The 2‐week group is shown in red, and the 4‐week group in blue.

Furthermore, the overall cumulative length of the newly formed vessel network was calculated (1544 ± 685 mm after 4 weeks and 1027 ± 628 mm after 2 weeks; n.s.). As most of the newly formed vessels displayed a radius of between 5 and ≈50 µm, we calculated the corresponding cumulative vessel length in both groups. We were able to demonstrate that most of the newly formed vessels displayed a radius within a range of 5–50 µm, but the cumulative length of the vessel network did not differ in this interval (947 ± 558 mm after 2 weeks vs 1495 ± 669 mm after 4 weeks; n.s.) (**Figure**  **9**).

Another parameter gathered from the XRM data is the volume of the vascular network. Calculating the volume of the newly formed vessels, there is a trend toward a larger newly formed network in a radius range of 5–50 µm (1.16 ± 0.56 mm^3^ after 4 weeks vs 0.74 ± 0.35 mm^3^ after 2 weeks; n.s.).

## 3 Discussion

Biofunctionalization of biomaterials has become an important tool in tissue engineering applications. Tailored functionalization allows the properties of materials to be better adapted to desired application areas. This can, for example, improve the stability of constructs or their vascularization. For instance, the addition of RGD motifs to spider silk hydrogels has improved the stability of the constructs over a period of 4 weeks.^[^ ^17^ ^]^ Additionally, angiogenesis was promoted by the RGD motifs. When methacryloyl was added to gelatin (so‐called GelMA), degradation could be decelerated.^[^ ^11a^ ^]^ Likewise, the unpredictable degradation rate of alginate can be rendered controllable with oxidation to ADA.^[^ ^18^ ^]^ Moreover, by coupling ADA hydrogels to gelatin, the biointeraction of the hydrogel can be adjusted.^[^ ^10^ ^]^ Apart from gelatin, laminin can also be used to functionalize gels in order to improve cell migration and angiogenesis.^[^ ^19^ ^]^ Laminin offers various binding sites such as RGD, collagen, and others, which mimic the ECM and therefore enhance the biocompatibility of materials.^[^ ^20^ ^]^ Apart from increasing biocompatibility, vascularization can also be promoted by biofunctionalization. It has already been demonstrated that with covalent bonding of the neuropeptide substance P, angiogenesis can be induced.^[^ ^21^ ^]^ Another way to enhance vascularization is with the addition of signaling molecules. In this process, known molecules such as vascular endothelial growth factors (VEGFs) and fibroblast growth factors (FGFs) can be used to stimulate new blood vessel formation.^[^ ^22^ ^]^

It has already been shown that vascularization can be positively influenced by cellularization.^[^ ^16b^ ^]^ Thus, cellularization is another option to enhance the vascularization of biomaterials.^[^ ^23^ ^]^ This was also achieved by co‐cultivation of fibroblasts and endothelial cells (HUVECs) demonstrating good cell migration and proliferation in ADA‐GEL.^[^ ^24^ ^]^ Moreover, Ruther and colleagues engineered vascular‐like structures with the use of ADA‐GEL, thus providing a starting point for the generation of bioartificial vessels.^[^ ^24^ ^]^ On the other hand, there is also the possibility to surgically induce intrinsic vascularization.^[^ ^25^ ^]^ For this purpose, Tanaka et al. compared different techniques.^[^ ^26^ ^]^ A distinction is made between the AV bundle and an AV loop. The AV bundle consists of a distal ligated artery with its venae comitantes that is passed through a biomaterial in parallel. In the AV loop model, an artery is connected to a vein by means of a vein graft, and the resulting loop is placed into the biomaterial. The AV loop has been shown to produce more vessels and new connective tissue, i.e., the vascularization of biomaterials works faster and better.^[^ ^26^ ^]^

In the present study, the principle of the AV loop also proved to be effective. It was demonstrated that in the ADA‐GEL‐hTG matrix, the percentage of connective tissue per construct area was significantly higher after 4 weeks with 14.93%, than within GelMA (3.44%), as tested in a former AV loop study.^[^ ^14^ ^]^ Distler and colleagues have already demonstrated that cross‐linking ADA‐GEL with transglutaminase allows for fine‐tuning toward better cell adhesion, cytocompatibility, and slower degradation of the required properties.^[^ ^14^ ^]^ The isopeptide binding of gelatin biomolecules by transglutaminases increases the constructs' stiffness, reducing swelling and degradation rates while maintaining high biocompatibility. In terms of biodegradation based on the remaining construct size after 4 weeks, the ADA‐GEL‐hTG matrix displayed faster biodegradation compared to former AV loop studies using scaffolds such as GelMA or recombinant spider silk ink. More precisely, 55% of the original GelMA and 68.5% of the original spider silk ink were still present in comparison to 18.4% ADA‐GEL‐hTG.^[^ ^11^, ^17^ ^]^

Fibrin is a Food and Drug Administration (FDA) ‐approved biomaterial that is widely used in clinical practice. The biodegradation of fibrin gel was investigated in a previous AV loop study revealing a very rapid biodegradation after 4 weeks. A direct comparison shows that significantly more of the ADA‐GEL‐hTG matrix remained after 4 weeks compared to the fibrin gel (18.4 vs 4.8%).^[^ ^27^ ^]^ In comparison with an earlier AV loop study, in which an ADA‐GEL hydrogel matrix with a lower polymer content (2.5/2.5% wv) was used, the increased polymer content (3.75/7.5%) and a higher degree of cross‐linking have no influence on biodegradability after 4 weeks (14.6 ± 3.66 mm^2^ vs 11.02 ± 1.76 mm^2^).^[^ ^13a^ ^]^

Regarding biocompatibility, the absence of foreign body giant cells and the equal proportion of pro‐ (M1) and anti‐inflammatory (M2) macrophages is a strong indicator for the low immunogenicity of the ADA‐Gel‐hTG.^[^ ^28^ ^]^ This supports the findings of previous in vivo studies with other ADA‐GEL compositions.^[^ ^13^, ^29^ ^]^ The presence of CD68‐positive cells, since they are found almost exclusively in the de novo formed tissue and at the interface with the matrix, could indicate that they are significantly involved in the formation and assembly of the connective tissue.

However, it was found that new vessel formation could be significantly influenced by the higher ADA‐GEL (3.75% ADA – 7.5% GEL) and higher cross‐linking degree when compared with a former ADA‐GEL study using lower polymer concentrations (2.5/2.5% wv). More precisely, the number of vessels in the current ADA‐GEL‐hTG study is already as high after 2 weeks as in the previous ADA‐GEL study after 4 weeks (24.5 ± 14.48 vs 31 ± 24).^[^ ^13a^ ^]^ After 4 weeks, about twice as many blood vessels were found in the current ADA‐GEL‐hTG study as in the previous ADA‐GEL study (70.25 ± 52.11 vs 31 ± 24). This makes the ADA‐GEL‐hTG hydrogel more suitable for possible transplantation of cells because they can be better supplied by faster vascularization. This result suggests that the increased polymer concentration and the higher degree of cross‐linking due to the addition of hTG have a positive effect on the improvement of vascularization. Compared to other leading biomaterials tested in the AV loop model, such as fibrin gel or recombinant spider silk matrices, a slightly lower number of vessels per cross‐section was found in the ADA‐GEL‐hTG matrix (107 ± 25 vs 99 ± 52 vs 70.25 ± 52.11).^[^ ^30^ ^]^

On the other hand, there was a significant increase in the number of blood vessels after 4 weeks compared to a previous AV loop study in which GelMA was used. Here, a fourfold increase in the number of newly formed blood vessels was found in in the ADA‐GEL‐hTG group compared with GelMA (70 ± 52 vs 16 ± 9).^[^ ^11a^ ^]^

Even though the results are promising, and the model is highly standardized, it is important to bear in mind that reliance on the rat AV loop model may not fully replicate the complexity of biocompatibility and vascularization in humans.

Regarding histology, there are some limitations with respect to quantitative analysis when assessing vessel formation. Since histology only depicts one or a few cross‐sections of a construct, X‐ray microscopy (XRM) is a more holistic approach for visualizing the entire vascular network. The high resolution of  5.9 µm voxel^−1^ enables the detection of small capillaries (≤10 µm diameter), as well as arterioles and venules (≤100 µm diameter). Although not statistically significant in histology, we observed more vessels per cross‐section after 4 weeks compared to the 2‐week group. Concordant with histology, we were able to prove vascularization of the ADA‐GEL‐hTG constructs. While histology only allows a limited quantification of angiogenesis per cross‐section, XRM allows a complete analysis of the AV loop. In this regard, the cumulative vessel length allows the quantification of all newly built vessels using XRM. In our study, we were able to detect a cumulative vessel length of 947 ± 558 mm after 2 weeks and 1495 ± 669 mm after 4 weeks. Moreover, XRM revealed that the vessel network matured as the vessels from the 4‐week group displayed a statistically significant larger radius compared to the 2‐week group. XRM data evaluation with specialized software such as XamFlow allows not only the analysis of 2D parameters such as the network length but also gives insight into 3D properties like the vessel network volume. This image can also be used to observe the quality or maturation of the vascular network over time. However, the XRM technique has also some limitations. The detection of the newly formed vessels is highly dependent on the complete perfusion of the specimen and the contrast agent. As contrast agents such as Microfil (used in this study) are characterized by a high viscosity, increasing with perfusion time, there is a risk that parts of the vessel network are not perfused at all. This can result in gaps or drop‐like features in the image that have to be excluded from the evaluation. In our study, we had to exclude one specimen from XRM analysis due to insufficient perfusion. For future XRM studies, a suitable contrast agent with a lower viscosity must be used in order to allow a complete perfusion of the specimen.

## 4 Conclusion

In this study, we were able to demonstrate the good biocompatibility of ADA‐GEL‐hTG hydrogels in vivo. In comparison with previous studies, the additional enzyme cross‐linker hTG and the elevated polymer concentration resulted in an enhancement of the angiogenic potential of the ADA‐GEL‐hTG matrix. This was accomplished without any deleterious effects on the processes of tissue formation and biodegradation. Furthermore, XRM microscopy has been shown to be an effective technique for more precise analysis of angiogenesis. The newly formed 3D vascular network can be reconstructed and subjected to quantitative analysis using XRM microscopy.

## 5 Experimental Section

### ADA‐GEL‐hTG Production

Alginate dialdehyde (ADA) was prepared by partial oxidation of sodium alginate Vivapharm PH163S2, which was approved as a pharmaceutical excipient. 1.337 g of sodium (meta) periodate (NaIO_4_ – BioUltra, ≥99.5%) was used as an oxidizing agent per 10 g of sodium alginate.^[^ ^13c^ ^]^

The exact procedure of the oxidation process was conducted as previously described.^[^ ^13c^ ^]^ For the preparation of human transglutaminase (hTG), Fibrogammin (200 U mL^−1^) was activated using CaCl_2_ (2.5 mm) and thrombin (7.5 U mL^−1^) overnight at 37 °C and stored at −80 °C until use. For the preparation of ADA‐GEL‐hTG, 7.5% (w/v) of ADA and 15% (w/v) of GEL were each dissolved by stirring at 37 °C for each implantation. After mixing equal volumes of the stock solutions, hTG was added to a final concentration of 20 U mL^−1^ and stirred for 10 min at 37 °C. Then, the resulting ADA‐GEL‐hTG hydrogel was sterile‐filtered to a final concentration of 3.75%ADA‐7.5%GEL.

### Arteriovenous Loop Surgery

**Figure 10:**
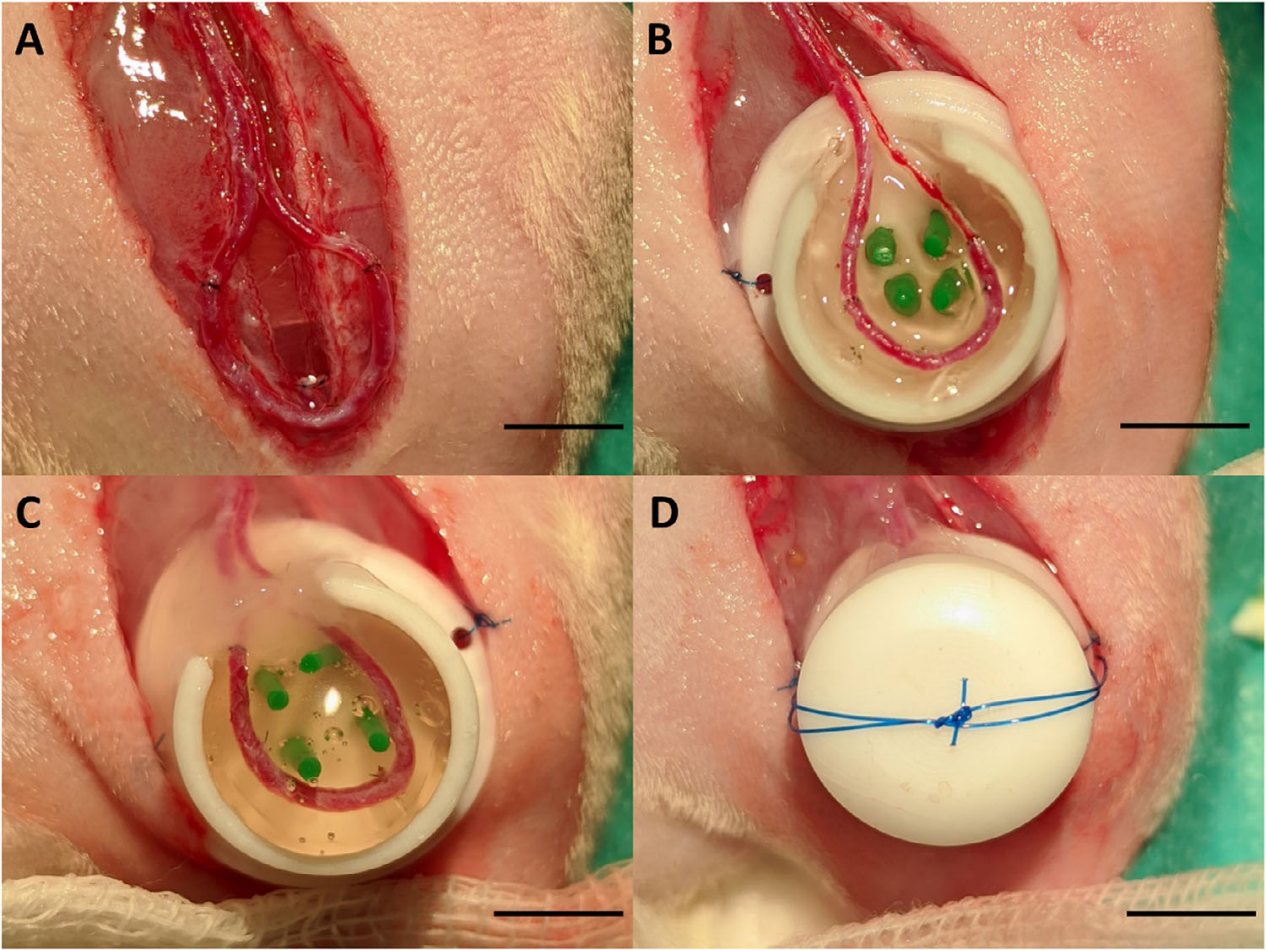
Arteriovenous (AV) loop operation. After the microsurgical anastomosis of the AV loop A), the loop was placed on top of the ADA‐GEL‐hTG hydrogel in the PTFE chamber B), covered with another layer of hydrogel C) and the chamber was closed with a lid D). Afterward, the skin was closed over the chamber. Scale bar = 5 mm.

The animal experiments were approved by the Animal Care Committee of the University of Erlangen and the Government of Mittelfranken (AZ 55.2‐2532‐2‐763) and were carried out according to EU Directive 2010/63/EU. For the AV loop surgeries, 6 male Lewis rats per group were operated on. Under anesthesia, an incision was made in the right groin of the rats, the saphenous artery and vein were dissected, and a vein graft was harvested. Thereafter, an arteriovenous loop (AV loop) was established between the left saphenous artery and vein using a vein graft with 11‐0 Ethilon (Ethicon, New Jersey, USA). The round Polytetrafluoroethylene (PTFE) chamber (inner diameter: 10 mm, height: 6 mm) was half‐filled with ADA‐GEL‐hTG and cross‐linked using 100 mm CaCl_2_ for 10 min. Thereafter, the PTFE chamber containing the ADA‐GEL‐hTG hydrogel was washed with Hanks' Balanced Salt Solution (HBSS; Sigma–Aldrich) and placed into the left groin. After positioning the AV loop in the PTFE chamber, the chamber was completely filled with ADA‐GEL‐hTG hydrogel, crosslinked, and washed as described above (**Figure** **10**). After the chamber was closed and fixed, the skin was sutured. The animals received pain medication and antibiotics for one week postoperatively, adjusted to body weight.

### Explantation Procedure

After 2 and 4 weeks of implantation, the animals were perfused with a contrast agent, which provides information about newly formed vessels during X‐ray microscopy (XRM) analysis. Under anesthesia, the animals were first perfused systemically with warm heparin solution via the aorta. Thereafter, the contrast agent Microfil (Flow Tech, Inc., Connecticut, USA) was applied for this purpose according to the manufacturer's instructions. Briefly, 8 mL of Microfil was mixed with a 10 mL diluent and preheated at 37 °C. Right before use, a 5% MV curing agent was added. The Microfil was allowed to cure overnight at 4 °C until removal of the chamber. The explanted construct was fixed in Histofix 4% (Carl Roth GmbH + Co. KG, Karlsruhe, Germany) for 24 h and then stored in HBSS until further use.

### X‐Ray Microscopy (XRM) Imaging

XRM scans were conducted with a Zeiss XradiaVersa 620 (Carl ZeissX‐ray Microscopy Inc., Pleasanton, CA, USA), equipped with a high framerate CMOS detector. Each tomography consisted of 1601 projection X‐ray radiographs (field of view: 12 mm) recorded at different angles. Magnification relied on the geometric magnification of the cone beam. The single projections were reconstructed by proprietary software (Zeiss XMReconstructor, Carl Zeiss X‐ray Microscopy Inc., Pleasanton, CA, USA) with a filtered back‐projection algorithm. The resulting volumetric images had a 3D isotropic voxel size of 5.9 µm. Data visualization and quantification were performed with XamFlow (Lucid Concepts AG, Zürich, Switzerland). Binary images, created by grey value thresholding and including the main vessel, were the basis of thickness, length, volume, and radius distribution calculations.

### Histological Staining

For the preparation of histological sections, the constructs were embedded in paraffin wax and cut into 3‐µm slices. H&E as well as α‐smooth muscle actin (α‐SMA) stains were prepared according to standard protocols. For the analysis of the constructs regarding biocompatibility, the constructs were examined for macrophages and large giant cells. Using CD68 staining, macrophages were detected. Therefore, the cross‐sections were deparaffinized, rehydrated, and incubated at room temperature for 20 min with pronase. Afterward, the cross‐sections were blocked with a blocking solution (Zytomed Systems GmbH, Berlin, Germany) and incubated with the primary antibody (anti‐CD68, BIO‐RAD, Hercules, USA) in a dilution of 1:300 overnight at 4 °C. After a washing step, the specimens were incubated with the secondary antibody (alkaline phosphatase‐labeled anti‐mouse antibody) for 30 min and stained with Fast Red TR/Naphthol AS (Sigma–Aldrich). Counterstaining was performed with hemalum.

Pro‐ (M1) and anti‐inflammatory (M2) macrophages were visualized using CD 86 and CD 163 immunostaining. Briefly, antigen retrieval was achieved by a boiling step. A blocking solution (Zytomed Systems GmbH, Berlin, Germany) was then added to the deparaffinized and rehydrated samples. Diluted anti‐CD86 (1:250, Abcam, Cambridge, USA) or anti‐CD163 (1:500, Leica Biosystems Inc., Buffalo Grove, USA) antibodies were then added and incubated overnight. Staining was performed with a second alkaline phosphatase‐conjugated anti‐mouse antibody (AP‐Polymer) and Fast Red TR/Naphthol AS substrate (Sigma). Hematoxylin was used for counterstaining.

For further analysis, images of the sections were either taken with the Olympus IX83 microscope (Olympus, Hamburg, Germany) using the cellSens Dimension V1.5 Software of Olympus, or scanned with the PANNORAMIC 250 Flash scanner (3DHISTECH, Budapest, Hungary), and the images were prepared using CaseViewer 2.4 (3DHISTECH).

For the evaluation of the construct and connective tissue area, the corresponding areas were outlined at the interface in GIMP 2.10 (GNU Image Manipulation Program) using the scissors select tool, and the area calculated in ImageJ 1.52 p (NIH, Bethesda, Maryland, USA). The area surrounding the main loop vessel was considered in the evaluation of the connective tissue area. For counting, the vessels with a defined lumen and filled with Microfil were counted manually in ImageJ.

### Statistical Analysis

One of six constructs in the 2‐week group and two of six constructs in the 4‐week group had to be excluded from statistical analysis due to thrombosis. Data were expressed as means ± standard deviation (SD). First, data was tested for normal distribution using Shapiro–Wilk test. For normally distributed data *t*‐test was carried out. Otherwise, the Mann–Whitney –U test was used. Analysis was performed using GraphPad Prism 9.00 (GraphPad Software, San Diego, USA). The graphical representation of the results showed the means ± SD. *p* values ≤ 0.05 were considered statistically significant. Significance was denoted by ^*^ *p* ≤ 0.05 and ^**^ *p* ≤ 0.01.

## Conflict of Interest

The authors declare no conflict of interest.

## Author Contributions

S.H.‐M. contributed to the investigation, visualization, writing of the original draft, and review and editing. J.H. contributed to the methodology and writing of the original draft. R.D. and A.R.B. contributed to funding acquisition, resources, supervision, and reviewing the draft. L.K. and S.C. contributed to the methodology and resources. S.P. contributed to the investigation, writing, and revision of the draft. R.R.K. and C.I.G. contributed to the investigation and revision of the draft. J.O. contributed to the revision of the draft. A.A. and R.E.H. contributed to conceptualization, funding acquisition, methodology, project administration, resources, supervision, and reviewing of the draft. D.S. contributed to the conceptualization, project administration, methodology, supervision, and writing and reviewing of the manuscript.

## Data Availability

The data that support the findings of this study are available from the corresponding author upon reasonable request.

## References

[1] N. Sari‐Chmayssem , S. Taha , H. Mawlawi , J.‐P. Guégan , J. Jeftić , T. Benvegnu , J. Appl. Phycol. 2016, 28, 1915.

[2] H. E. Thu , M. H. Zulfakar , S. F. Ng , Int. J. Pharm. 2012, 434, 375. 22643226 10.1016/j.ijpharm.2012.05.044

[3] a) C. H. Goh , P. W. Heng , E. P. Huang , B. K. Li , L. W. Chan , J. Antimicrob. Chemother. 2008, 62, 105; 18408234 10.1093/jac/dkn168

[4] D. Ji , J. M. Park , M. S. Oh , T. L. Nguyen , H. Shin , J. S. Kim , D. Kim , H. S. Park , J. Kim , Nat. Commun. 2022, 13, 3019. 35641519 10.1038/s41467-022-30691-z PMC9156673

[5] S. Shafei , M. Khanmohammadi , R. Heidari , H. Ghanbari , V. Taghdiri Nooshabadi , S. Farzamfar , M. Akbariqomi , N. S. Sanikhani , M. Absalan , G. Tavoosidana , J. Biomed. Mater. Res. A 2020, 108, 545. 31702867 10.1002/jbm.a.36835

[6] B. Sarker , T. Zehnder , S. N. Rath , R. E. Horch , U. Kneser , R. Detsch , A. R. Boccaccini , ACS Biomater. Sci. Eng. 2017, 3, 1730. 33429654 10.1021/acsbiomaterials.7b00188

[7] H. Liao , H. Zhang , W. Chen , J. Mater. Sci. Mater. Med. 2009, 20, 1263. 19184370 10.1007/s10856-009-3694-4

[8] E. K. Lisa Schöbel , R. Detsch , A. R. Boccaccini , Mater. Lett. 2023, 340, 134103.

[9] L. Sprenger , H.‐H. Lu , S. Trippmacher , U. Mansfeld , P. Milkin , L. Ionov , G. Papastavrou , A. R. Boccaccini , S. Salehi , ACS Appl. Mater. Interfaces 2024, 16, 44605. 39159061 10.1021/acsami.4c10751

[10] a) Z. Safari , S. Soudi , N. Jafarzadeh , A. Z. Hosseini , E. Vojoudi , M. Sadeghizadeh , Sci. Rep. 2019, 9, 11182; 31371773 10.1038/s41598-019-47413-z PMC6672002

[11] a) S. Heltmann‐Meyer , D. Steiner , C. Müller , D. Schneidereit , O. Friedrich , S. Salehi , F. B. Engel , A. Arkudas , R. E. Horch , Biomed. Mater. 2021, 16, 065004; 10.1088/1748-605X/ac1e9d 34406979

[12] E. Entekhabi , M. Haghbin Nazarpak , M. Sedighi , A. Kazemzadeh , Mater. Sci. Eng., C 2020, 107, 110362. 10.1016/j.msec.2019.110362 31761181

[13] a) D. Steiner , L. Lingens , L. Fischer , K. Köhn , R. Detsch , A. R. Boccaccini , T. Fey , P. Greil , C. Weis , J. P. Beier , R. E. Horch , A. Arkudas , Tissue Eng. Part A 2018, 24, 1320; 29652607 10.1089/ten.TEA.2017.0496

[14] T. Distler , K. McDonald , S. Heid , E. Karakaya , R. Detsch , A. R. Boccaccini , ACS Biomater. Sci. Eng. 2020, 6, 3899. 33463325 10.1021/acsbiomaterials.0c00677

[15] Z. Valnickova , J. J. Enghild , J. Biol. Chem. 1998, 273, 27220. 9765243 10.1074/jbc.273.42.27220

[16] a) O. O. Erol , M. Sira , Plast. Reconstr. Surg. 1980, 66, 109; 6156468 10.1097/00006534-198007000-00021

[17] D. Steiner , S. Winkler , S. Heltmann‐Meyer , V. T. Trossmann , T. Fey , T. Scheibel , R. E. Horch , A. Arkudas , Biofabrication 2021, 13, 045003. 10.1088/1758-5090/ac0d9b 34157687

[18] K. H. Bouhadir , K. Y. Lee , E. Alsberg , K. L. Damm , K. W. Anderson , D. J. Mooney , Biotechnol. Prog. 2001, 17, 945. 11587588 10.1021/bp010070p

[19] a) C. Mukherjee , S. Saleem , S. Das , S. C. Biswas , D. Bhattacharyya , J. Biosci. 2020, 45, 93; 32713856

[20] a) M. Aumailley , M. Gerl , A. Sonnenberg , R. Deutzmann , R. Timpl , FEBS Lett. 1990, 262, 82; 2318314 10.1016/0014-5793(90)80159-g

[21] M. Shafiq , Y. Jung , S. H. Kim , J. Biomed. Mater. Res. A 2015, 103, 2673. 25641807 10.1002/jbm.a.35400

[22] a) Q. He , Y. Zhao , B. Chen , Z. Xiao , J. Zhang , L. Chen , W. Chen , F. Deng , J. Dai , Acta Biomater. 2011, 7, 1084; 20977949 10.1016/j.actbio.2010.10.022

[23] A. Kengelbach‐Weigand , C. Thielen , T. Bauerle , R. Gotzl , T. Gerber , C. Korner , J. P. Beier , R. E. Horch , A. M. Boos , NPJ Regen. Med. 2021, 6, 49. 34413320 10.1038/s41536-021-00158-8 PMC8377075

[24] F. Ruther , T. Distler , A. R. Boccaccini , R. Detsch , J Mater. Sci. Mater. Med. 2018, 30, 8. 30594988 10.1007/s10856-018-6205-7

[25] T. Nariai , R. Suzuki , Y. Matsushima , K. Ichimura , K. Hirakawa , K. Ishii , M. Senda , Stroke 1994, 25, 1014. 8165672 10.1161/01.str.25.5.1014

[26] Y. Tanaka , K. C. Sung , A. Tsutsumi , S. Ohba , K. Ueda , W. A. Morrison , Plast. Reconstr. Surg. 2003, 112, 1636. 14578795 10.1097/01.PRS.0000086140.49022.AB

[27] A. Arkudas , G. Pryymachuk , T. Hoereth , J. P. Beier , E. Polykandriotis , O. Bleiziffer , R. E. Horch , U. Kneser , Tissue Eng. Part A 2009, 15, 2501. 19292678 10.1089/ten.tea.2008.0477

[28] a) T. L.už Božinovski , V. Todorović , I. Milošević , B. B. Prokić , V. Gajdov , K. Nešović , V. Mišković‐Stanković , D. Marković , J. Biomater. Appl. 2022, 36, 1111; 34607494 10.1177/08853282211046119

[29] a) U. Rottensteiner , B. Sarker , D. Heusinger , D. Dafinova , S. N. Rath , J. P. Beier , U. Kneser , R. E. Horch , R. Detsch , A. R. Boccaccini , A. Arkudas , Materials 2014, 7, 1957; 28788549 10.3390/ma7031957 PMC5453292

[30] a) T. A. Ahmed , E. V. Dare , M. Hincke , Tissue Eng. Part B Rev. 2008, 14, 199; 18544016 10.1089/ten.teb.2007.0435

